# Exploring gait automaticity and prefrontal brain activity during single and dual-task walking in aging and Parkinson’s disease

**DOI:** 10.1186/s12984-025-01864-w

**Published:** 2026-01-05

**Authors:** Alexander Kvist, Daniel S. Peterson, Lucian Bezuidenhout, Hanna Johansson, Franziska Albrecht, Urban Ekman, David Moulaee Conradsson, Erika Franzén

**Affiliations:** 1 Department of Neurobiology, Care Sciences and Society, Division of Physiotherapy, Karolinska Institutet, Stockholm, Sweden; 2 College of Health Solutions, Arizona State University, Phoenix, USA; 3 Department of Health and Rehabilitation Sciences, Division of Physiotherapy, Stellenbosch University, Cape Town, South Africa; 4 Women’s Health and Allied Health Professionals Theme, Medical Unit Allied Health Professionals, Karolinska University Hospital, Stockholm, Sweden; 5 Research and Development Unit, Stockholm Sjukhem Foundation, Stockholm, Sweden; 6 Department of Neurobiology, Care Sciences and Society, Division of Clinical Geriatrics, Karolinska Institutet, Stockholm, Sweden

**Keywords:** Dual-task, FNIRS, Gait, Walking, Cognitive-motor interference, Automaticity

## Abstract

**Background:**

Walking while performing a concurrent cognitive task leads to cognitive-motor interference, resulting in slower and more variable gait. This is particularly the case in Parkinson’s disease (PD), where dual-task situations exacerbate walking impairments, increasing fall risk and reducing quality of life. Cognitive-motor impairment has been linked to excessive attentional demands due to reduced locomotor automaticity. Neuroimaging studies suggest over-reliance on prefrontal resources potentially reflecting compensatory mechanisms. However, few studies link automaticity, performance, and cognitive capacity to prefrontal activity, particularly in PD. In older adults (OA) and people with PD, this study aims to: (1) describe dual-task effects and prefrontal cortical activity during walking with and without a dual-task, (2) determine the connection between prefrontal cortical activity and step time variability as a measure of gait automaticity, (3) explore associations between prefrontal cortical activity and other measures of gait automaticity and prioritization and (4) investigate executive function as a potential moderator in the compensatory relationship.

**Methods:**

Data from 44 OA and 37 people with PD walking with and without an auditory Stroop task were analyzed. Gait variables were measured using inertial measurement units, and prefrontal activity was assessed with functional near-infrared spectroscopy (fNIRS). Executive function was determined with a trail making test. Data analysis involved linear regression models to explore relationships between prefrontal activity, gait automaticity, and executive function.

**Results:**

Most participants had a cognitive priority trade-off when dual-tasking, and the OA group had more prefrontal activity compared to the PD group during single-task and dual-task walking. For PD there was a significant positive relationship between step time variability and prefrontal activity (β = 0.38, T = 6.26, p < 0.01), while OA had a relationship between age and prefrontal activity (β = 0.53, T = 2.33, p = 0.04). Secondary analyses showed relationships between prefrontal activity and dual-task cost of gait speed (β = 0.25, T = 2.90, p = 0.02) and Stroop response time (β = 0.27, T = 3.10, p = 0.01) in PD, but not in OA. No moderation effects were detected in the relationship between gait automaticity and prefrontal activity.

**Conclusions:**

In PD, loss of gait automaticity is linked to increased prefrontal activity, suggesting compensatory mechanisms. In OA, prefrontal activity during walking seems to be primarily age-related.

**Supplementary Information:**

The online version contains supplementary material available at 10.1186/s12984-025-01864-w.

## Background

Walking while performing a concurrent cognitive task gives rise to cognitive-motor interference, leading to poorer performance such as slower walking speed [2, 58], and increased gait variability [59]. While this is true in both aging [6] and various neurodegenerative diseases [43], people with Parkinson’s disease (PD) especially have been shown to exhibit walking impairments elicited by dual-task situations [46], relating to increased fall risk and poorer quality of life [34].

Although numerous studies have evaluated cognitive-motor interference while walking, the underlying causes of cognitive-motor interference remain incompletely understood. An influential early article suggested that a contributor to dual-task impairments and fall risk could be a “posture second” strategy, such that people with PD exhibit worse gait and balance while performing cognitive tasks during walking due to focusing on the cognitive task instead of maintaining balance [8]. However, later work did not consistently observe the posture-second strategy in people with PD [34].

Another proposed contributor to dual-task impairments is excessive attentional demands of gait due to reduced locomotor automaticity [12], where basal ganglia dysfunction in PD could lead to reduced locomotor automaticity [34]. This is supported by the observation that dual-task impairments among people with PD increase with cognitive load [26, 41] and are exacerbated by reduced executive function [68].

While many studies investigating cognitive-motor interference have been largely behavioral, neuroimaging studies of dual-tasking [46], including dual-task walking [33, 35, 49, 64], give a more detailed picture, indicating that the prefrontal cortex is involved in dual-task performance. Specifically, the indirect [28, 30] or executive locomotor [12, 38] pathway, involving the prefrontal cortex, may be relied upon more heavily in people with dual-task impairments due to both reduced locomotor automaticity and increased executive demands of the concurrent task. The over-reliance on prefrontal resources has been proposed to reflect a compensatory mechanism to maintain walking performance despite reduced locomotor automaticity [47].

Several theoretical models have been proposed to contextualize such compensatory activity [21]. In particular, the Compensation-Related Utilization of Neural Circuits Hypothesis (CRUNCH) model [53] was proposed to explain functional brain imaging observations of over activation in older adults compared to younger adults during cognitive and motor tasks, where compensation by upregulation or reorganization [11] of activity from automatic circuits to volitional prefrontal circuits during normal walking might explain increased dual-task interference. The CRUNCH model could also be applied to pathological aging [9] including PD [35], with an important additional observation of compensatory mechanisms breaking down at a certain tipping point of neural degeneration or task demand leading to substantially reduced performance on the gait and/or cognitive tasks [9].

To test compensation models, it is helpful to link brain activity to observed task performance. However, few studies have examined this link in PD. While systematic reviews show that several studies have investigated brain activity during walking [49] and dual-tasking in people with PD [33], a 2023 review of functional near-infrared spectroscopy (fNIRS) studies [60] found only 10 studies to have examined associations between walking performance and brain activity in PD. Looking more specifically at dual-task performance, a 2022 review [35] found only 4 papers (3 fNIRS, 1 functional magnetic resonance imaging) to have correlated dual-task performance to brain activity in PD. Findings have been mixed, but two studies [48, 65], found connections between prefrontal cortex activity and step time variability—an outcome suggested to reflect locomotor automaticity [24]. Other studies have found connections between prefrontal cortex activity and cognitive ability. For example, Maidan et al., [42] observed increases in prefrontal activation from single to dual-task related to executive function [42]. However, the directionality of these relationships were variable across studies. Together this work suggests a possible neural mechanism for dual-task impairments in people with PD—that reduced automaticity leads to increased cortical activation during gait to maintain performance, possibly connected to cognitive ability such as executive function. However, as noted above, given the small number studies investigating this specific topic [42, 48, 65], and their variable findings, additional work is necessary to establish evidence for the hypothesis that compensatory activity occurs in order to compensate for a reduced automaticity, and to assess the vigor of this proposed mechanism in older adults and people with PD.

To better understand prefrontal cortical activity during walking and dual-task walking in older adults and people with PD, this study aims to (1) describe dual-task effects and prefrontal cortical activity during walking and dual-task walking, (2) determine the connection between prefrontal cortical activity and step time variability as a measure of gait automaticity, (3) explore associations between prefrontal cortical activity and other measures of gait automaticity as well as prioritization, and (4) explore executive function as a potential moderator in the compensatory relationship.

## Material and methods

### Dataset

This study uses data from the “ParkMOVE” dataset that we collected between 2021 and 2023 [22]. Data from two groups were used: older adults and people with PD. For this dataset, experiments took place across two sessions at the uMOVE core facility, Karolinska University Hospital, Solna, Stockholm. During the experimental sessions, clinical tests of balance, disease severity and a neuropsychological test battery were performed, along with fNIRS measurement during a block-based complex walking protocol. Participants performed the fNIRS measurement during one first session, and clinical tests and neuropsychological tests during another with approximately one week between the sessions.

### Open lab notebook

Since this study uses an already existing dataset, the study is conducted as an open lab notebook study [39], [55] where planned analyses, performed analyses, robustness and sensitivity analyses are documented. The notebook details how the data has been accessed, decisions and updates during data processing, statistical model validation, and all code used to generate tables and figures. The notebook can be found in a version-tracked repository at: https://alkvi.github.io/fnirs_stroop_study

### Experimental procedure

Clinical tests of balance were performed for the older adult and PD groups with the Mini-Balance Evaluation Systems Test (Mini-BESTest) [17] and disease severity for the PD group with the Movement Disorder Society-sponsored revision of the Unified Parkinson’s Disease Rating Scale (MDS-UPDRS) [25].

The neuropsychological test battery comprised the following tests: The Color-Word Interference Test (CWIT) part III from the Delis-Kaplan Executive Function System (D-KEFS) [15], Verbal Fluency part I-III (from D-KEFS), Trail Making Test (TMT) part II and IV (from D-KEFS) and Ray Auditory Verbal Learning Test (RAVLT) [56].

The walking protocol (fully described in [22]) used during the fNIRS measurement contained blocks of straight walking (Walking ST), standing still while performing an auditory Stroop task (Standing ST) and straight walking while performing the auditory Stroop task (Walking DT). For the straight walking condition, participants were asked to walk straight at a self-selected speed to a cone 30 m from the starting cone and back. Block length was 20 s long, followed by 15 s of rest period to allow for a baseline measure. Each block condition was performed 6 times (e.g., 6 blocks of walking straight) with the time being approximately 12 min to complete the protocol.

The auditory Stroop task consisted of the Swedish words for high and low in a congruent or incongruent high and low pitch. Words were presented to the participants through wireless headphones. Participants were instructed to respond verbally, as fast and correctly as possible, to the corresponding pitch irrespective of the words presented. The responses were recorded to analyze task accuracy and reaction time. During each block with an auditory Stroop task, a total of 7-word prompts of high or low were presented in a predetermined randomized order. Participants were instructed to pay equal attention to both tasks when dual-tasking.

The fNIRS system used was a NIRSport2 (NIRx) with 8 sources and 8 detectors, with 8 short-separation detection channels to allow for removing superficial blood flow changes in the signal. The optodes transmitted light at 760 and 850 nm, and the sampling frequency was 10 Hz. Data was captured using Aurora (NIRx) (v.1.4). The optodes were fitted to a cap according to the international 10–20 system and placed over the prefrontal area (supplementary Fig. 1). Caps were chosen in accordance with the head sizes of participants.

Gait parameters (e.g., step time and walking speed) were collected using three wireless inertial sensors (Opal, APDM Inc.) positioned over the lumbar and on top of each foot near the ankle. Raw data in each block was analyzed in the python Gaitmap library [37]. Step time variability was calculated based on the standard deviation of left and right steps according to Galna et al., [23]. Two strides were excluded from the start and end of each block to get steady state gait [44, 61].

Outliers (identified with box plots as observations over or below 1.5 interquartile range) in gait and auditory Stroop variables that could be traced via protocol observation comments to unexpected behavior in the protocol (e. g., stopping during a walking block) were excluded from analysis. Excluded and missing subjects are detailed in supplementary document 1.

### Data analysis

Analysis of fNIRS data was performed in the MATLAB NIRS AnalyzIR toolbox (forked version; see Data availability for details) [54]. The raw optical density fNIRS data was converted into oxygenated hemoglobin (HbO2) and deoxygenated hemoglobin (HbR) using the modified Beer-Lambert law [16] with the differential path-length factor (DPF) dependent on age [57].

Analyses used a combined form of HbO2 and HbR, in terms of correlation-based signal improvement (CBSI) [13]. While often used as a movement artifact correction method, this can be considered as a version of the HbO2 scaled for anticorrelation with the HbR, incorporating information from both into one signal.

The first level (subject level) analysis employed a general linear model (GLM), using pre-whitening and an autoregressive model (AR-IRLS) [5] to reduce systemic physiology and motion-induced artifacts. Short-separation channels were used as regressors to further filter out physiological noise. A canonical hemodynamic response function (HRF) was assumed.

The second level (group level) analysis used linear regression models to investigate the effects of covariates on measured prefrontal cortex activity. Models were evaluated in each group separately. From the group-level models, model summary statistics were obtained (R squared, F statistic, p value). Model outputs were used in a region of interest (ROI) analysis across the prefrontal cortex. This ROI included all channels, except short channels.

### Statistical analysis

Normality of demographic and task performance data was assessed with the Shapiro–Wilk normality test and visually with q-q plots in R (v4.2.2) [51]. Data was then compared between groups using the arsenal (v3.6.3) package. Comparison was done with the Kruskal–Wallis test with alpha level set at 0.05.

For aim 1 of describing dual-task effects and prefrontal cortex activity, dual-task effects (DTEs) were calculated to assess cognitive-motor interference:

$$DTE = \frac{{dual - task\,performance - single - task\,performance}}{{single - task\,performance}} \times 100\%$$

In particular, the DTE on walking speed (motor dual-task effect) and Stroop reaction time (cognitive dual-task effect) were calculated, with the convention that a negative DTE indicates a deteriorated performance during dual-task compared to single-task. To align with the convention, a negative sign was inserted where a higher value indicates a poorer performance (in this case reaction time). A visualization of channel-level contrast statistics was also computed to compare the prefrontal cortex activity in the two groups directly using the NIRS toolbox *ttest* function, with the group level model only using task condition as a covariate.

For aim 2 of examining the connection between prefrontal cortical activity and step time variability as a measure of gait automaticity, a linear regression model was used. The model related step time variability to prefrontal activation, controlling for age and disease severity. Disease severity was quantified by calculating the MDS-UPDRS III motor score from the MDS-UPDRS, and chosen after comparing the Akaike Information Criterion (AIC) for MDS-UPDRS III motor score and disease duration. Step time variability was chosen as a measure of gait automaticity because it can be argued [24] that it directly links a motor deficit to an impairment in motor automaticity [67]. A larger step time variability indicates a loss of gait automaticity. The following model was used, with MDS-UPDRS motor score only for the PD group:

$$\beta \sim - 1 + condition + step\,time\,variability + age + \left( {MDS - UPDRS\,motor\,score} \right)$$

For visualization purposes, the correlation between subject-level prefrontal cortex activity and step time variability was plotted in R using ggpmisc *stat_correlation* [3].

For aim 3, analyses explored relations between prefrontal cortex activity and two other measures of automaticity as well as priority. Dual-task costs (DTCs) were used as measures of automaticity, where a larger cost indicates a larger dual-task performance deterioration and therefore less automaticity (i.e., the negative DTEs). Relations to motor or cognitive prioritization was also explored, where priority was calculated as *priority* = *DTC_motor—DTC_cognitive*. The linear models used were:

$$\beta \sim -1+condition+DTC\_motor+age+\left(MDS-UPDRS\, motor \,score\right)$$

$$\beta \sim -1+condition+DTC\_cognitive+age+\left(MDS-UPDRS \,motor \,score\right)$$

$$\beta \sim -1+condition+priority+age+\left(MDS-UPDRS\,motor\, score\right)$$

As a last aim (aim 4), we tested the assumption that dual-task impairments are exacerbated by reduced executive function by correlating a measure of executive ability (trail making test (TMT) IV) to dual-task costs. The TMT IV assesses task-set-shifting which has certain neuro-cognitive overlaps with dual-tasking [66]. We also tested if executive ability acts as moderator on the relationship between gait automaticity and prefrontal activity, additionally controlling for years of education:

$$\begin{gathered} \beta \sim - 1 + condition + step\,time\,variability \times TMT\_IV \hfill \\ + age + education + \left( {MDS - UPDRS\,motor\,score} \right) \hfill \\ \end{gathered}$$

To account for multiple comparisons in each aim, the p-values of the linear models were adjusted using Benjamini–Hochberg false discovery rate (FDR) correction.

Model residuals were checked to see if they fulfill assumptions of linearity, homoscedasticity and normality of residuals (available in the open lab notebook). Residual-leverage plots and the RemoveOutlierSubjects function in the NIRS toolbox were also used to test for robustness against influential outliers.

## Results

### Participants

After excluding participants with missing fNIRS data, step time variability, or disease severity, there remained 44 subjects from the older adult group (60–85 yrs, mean 69 ± 7 yrs) and 37 subjects from the PD group (60–91 yrs, mean 69 ± 7 yrs). An additional PD participant missed reaction time data and an additional older adult participant lacked dual-task cost on walking speed and were not used in corresponding linear models in aim 3.

Compared to the older adult group (Table 1), the PD group had worse balance (Mini-BESTest score 23 vs 25, p < 0.01), along with a lower walking speed (1.13 m/s vs 1.23 m/s, p = 0.02) and higher step time variability (24.18 ms vs 18.36 ms, p = 0.01) during dual-task walking, and a greater dual-task cost on walking speed (4.35% vs 0.92%, p < 0.01).

**Table 1:** Demographics, cognitive and motor performance data

Measure, mean (SD) unless otherwise stated	OA (N = 44)	PD (N = 37)	Total (N = 81)	p value
Gender, female, N, (%)	19 (43.2%)	16 (43.2%)	35 (43.2%)	1.00
Age, yrs	69 (7)	69 (7)	69 (7)	0.90
Education, yrs	15 (3)	16 (2)	15 (2)	0.28
Weight, kg	74 (14)	76 (17)	75 (16)	0.82
Height, cm	174 (10)	173 (9)	173 (10)	0.85
MDS-UPDRS III score	N/A	25.38 (9.31)	N/A	N/A
Hoehn & Yahr stage (counts)	N/A	I: 2, II: 20, III: 14, IV: 1	N/A	N/A
Levodopa equivalent daily dose*, mg/day	N/A	452.87 (228.46)	N/A	N/A
Mini-BESTest score	25 (2)	23 (3)	24 (3)	** < 0.01**
TMT II, s	37.31 (16.92)	58.98 (32.21)	47.21 (27.18)	** < 0.01**
TMT IV, s	80.24 (32.99)	112.33 (71.69)	94.64 (55.87)	**0.02**
TMT IV – TMT II, s	44.02 (31.61)	55.42 (59.66)	49.14 (46.36)	0.38
Walking speed (ST), m/s	1.24 (0.22)	1.19 (0.18)	1.21 (0.20)	0.10
Walking speed (DT), m/s	1.23 (0.23)	1.13 (0.20)	1.18 (0.22)	**0.02**
Step time variability (ST), ms	18.02 (6.83)	22.88 (17.59)	20.24 (13.04)	0.83
Step time variability (DT), ms	18.36 (14.91)	24.18 (15.82)	20.98 (15.50)	**0.01**
DT cost walking speed, %	0.92 (4.18)	4.35 (5.54)	2.46 (5.11)	** < 0.01**
DT cost step time variability, %	−4.97 (91.24)	−12.51 (43.31)	−8.36 (73.33)	0.09
Accuracy (ST), %	98.39 (3.81)	95.69 (8.43)	97.14 (6.48)	0.11
Reaction time (ST), s	1.09 (0.19)	1.07 (0.18)	1.08 (0.19)	0.65
Accuracy (DT), %	98.12 (4.01)	94.53 (10.85)	96.46 (8.09)	0.09
Answer time (DT), s	1.05 (0.20)	1.04 (0.19)	1.04 (0.19)	0.88
DT cost reaction time, %	−4.19 (5.58)	−2.58 (7.38)	−3.45 (6.48)	0.38

^*^Missing data for N = 6. Bold indicates p < .05. ST: Single-task; DT: Dual-task; TMT: Trail making test; MDS-UPDRS: Movement Disorders Society-Unified Parkinson’s Disease Rating Scale

### Dual-task effects and prefrontal cortex activity during task conditions

**Fig. 1:**
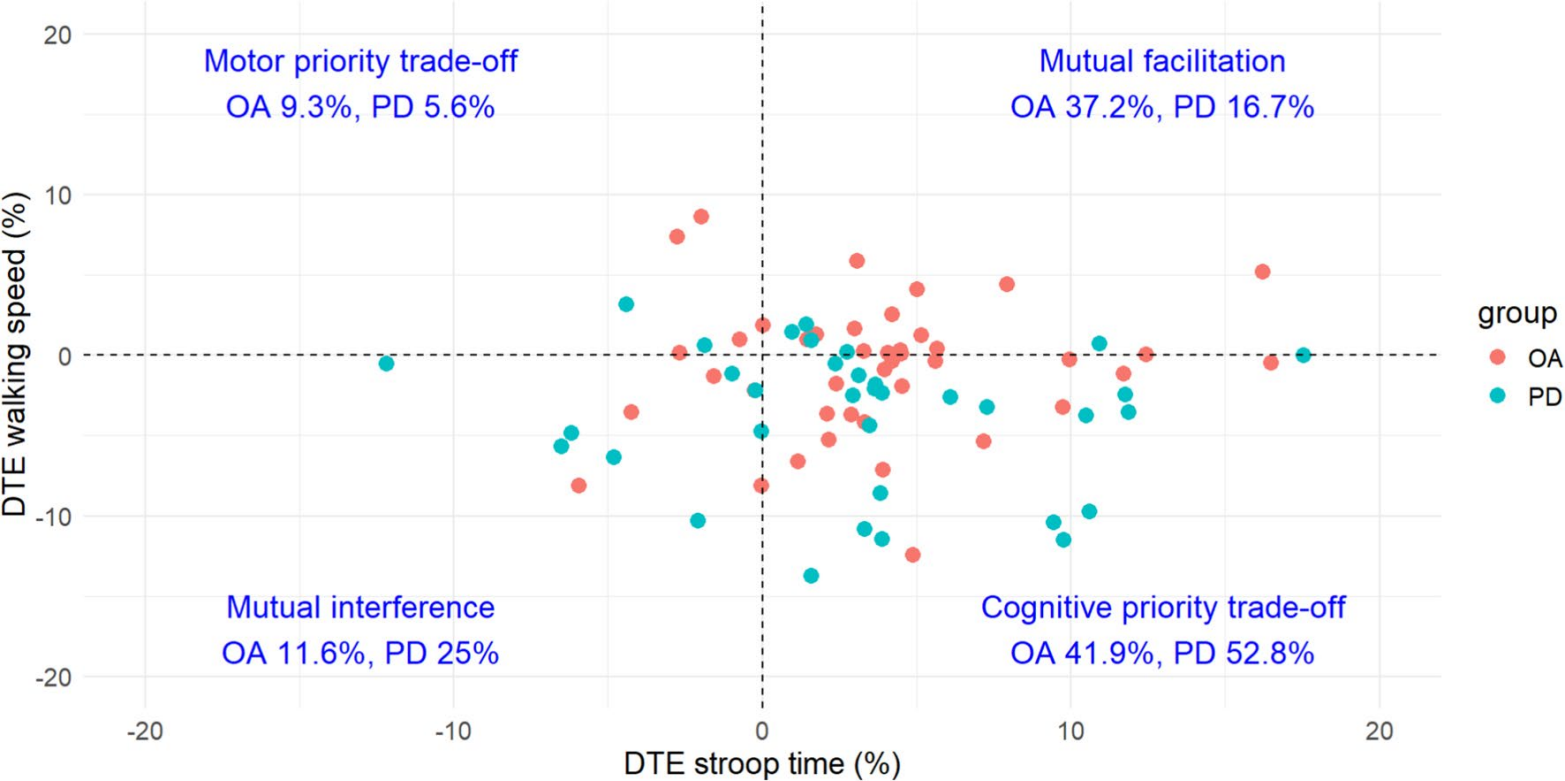
Quadrant of dual-task effects. DTE: Dual-task effect; OA: Older adults; PD: Parkinson’s disease

Cognitive-motor interference and prioritization during dual-tasking are illustrated in Fig. 1: 41.9% of older adults and 52.8% of participants with PD had a cognitive priority trade-off when dual-tasking, followed by mutual facilitation (37.2% older adult, 16.7% PD).

**Fig. 2:**
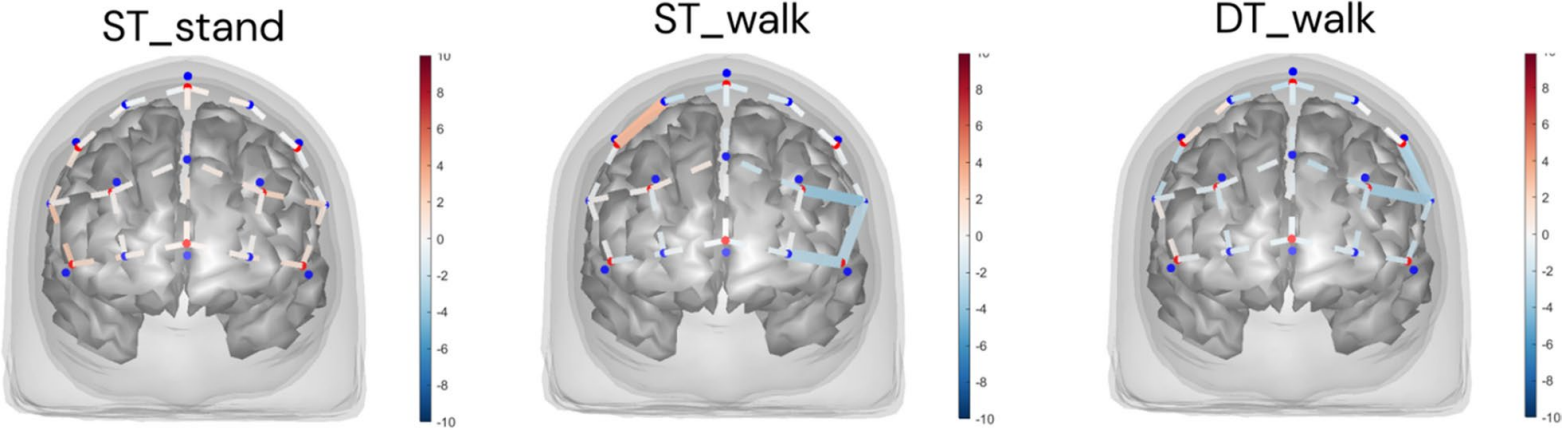
Channel-level contrast in each experimental condition between OA and PD, contrast defined as PD–OA. Color scale indicates T value. Red indicates positive contrast (PD > OA) and blue negative (PD < OA). Solid lines indicate channels with significant differences (FDR p < 0.05). ST: Single-task; DT: Dual-task; OA: Older adults; PD: Parkinson’s disease

Comparison of prefrontal cortex activity during the experimental tasks revealed no difference in activity between the older adult and PD group in the standing auditory Stroop condition (Fig. 2). During single-task and dual-task walking, the older adult group had several channels in the left prefrontal cortex indicating more activity compared to the PD group. During single-task walking, the older adult group had more activity in channels AF7-F5, AF7-Fp1 and AF3-AF5. During dual-task walking, the older adult group had more activity in channels F3-F5 and AF3-AF5. Prefrontal cortex activity for each group can be found in supplementary Fig. 2 A full table of channels and associated statistics can be found in supplementary Table 1.

### Associations between step time variability and prefrontal cortex activity

**Fig. 3:**
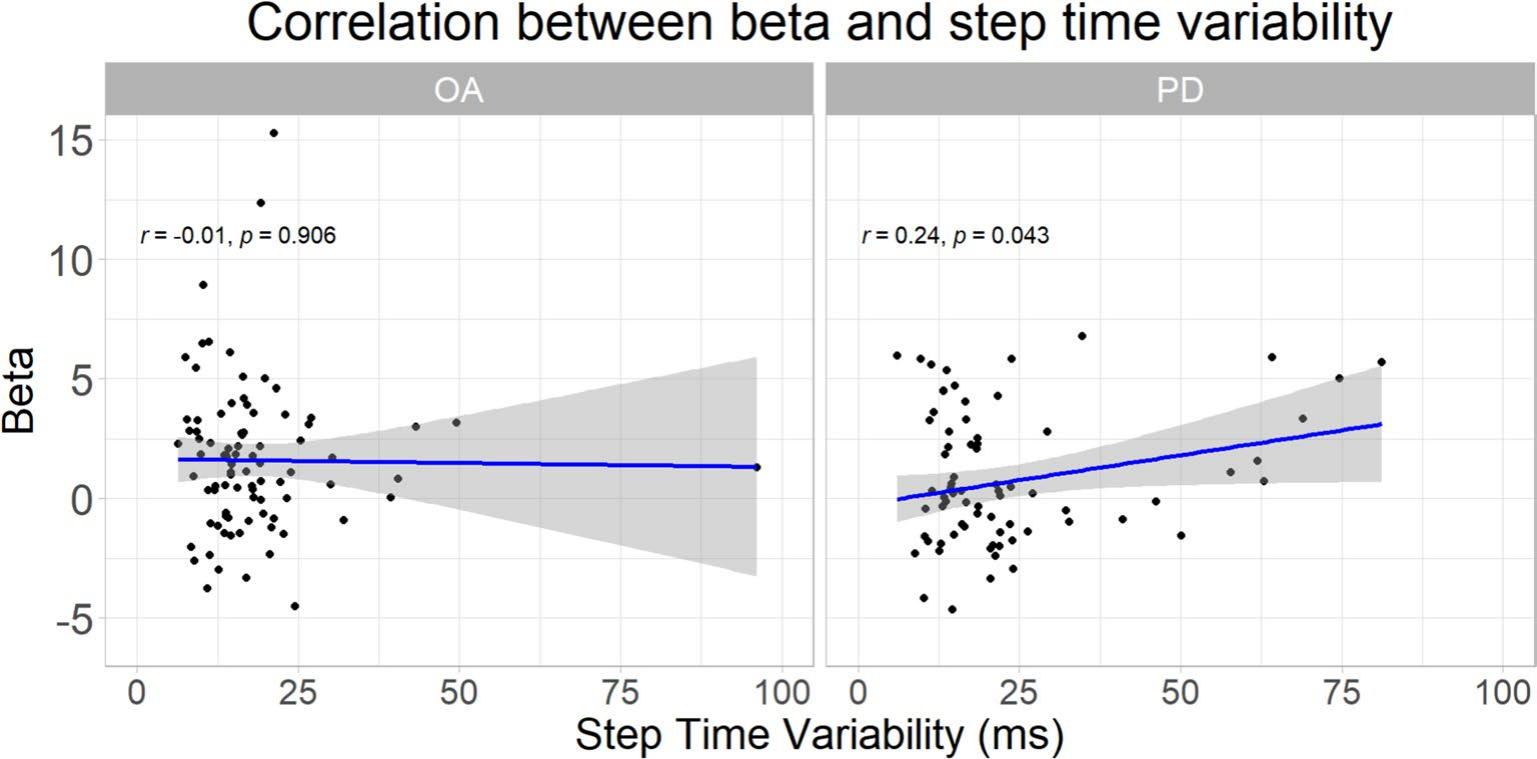
Correlations between subject-level prefrontal cortex activity and step time variability during single- and dual-task walking. OA: older adults; PD: Parkinson’s disease

There was a significant relationship between a higher step time variability and a higher prefrontal activity for PD (β = 0.38, T = 6.26, p < 0.01) but not older adult (Table 2). For older adult, there was instead a significant relation between older age and a higher prefrontal activity (β = 0.53, T = 2.33, p = 0.04). Bivariate correlations between step time variability and subject-level prefrontal cortex activity in each group are shown in Fig. 3 (older adult no correlation r = −0.01, PD positive correlation r = 0.24).

**Table 2:** fNIRS linear model results for aim 2: relating prefrontal cortex activity to step time variability

Covariate	Beta	SE	DF	T	p	FDR p	N
Group: OA							
Model: R square = 0.114, R square (adjusted) = 0.0796, F = 3.255, p < 0.001							
ST_stand condition	−0.27	0.14	127	−1.92	0.057	0.089	44
ST_walk condition	0.94	0.15	127	6.26	< 0.001	** < 0.001**	44
DT_walk condition	1.40	0.16	127	8.51	< 0.001	** < 0.001**	44
Age	0.53	0.23	127	2.33	0.021	**0.039**	44
Step time variability	−0.17	0.14	127	−1.23	0.221	0.243	44
Group: PD							
Model: R square = 0.144, R square (adjusted) = 0.0952, F = 2.941, p < 0.001							
ST_stand condition	0.22	0.14	105	1.59	0.116	0.141	37
ST_walk condition	0.27	0.15	105	1.82	0.071	0.098	37
DT_walk condition	0.44	0.16	105	2.67	0.009	**0.024**	37
Age	0.24	0.26	105	0.94	0.351	0.351	37
Step time variability	0.38	0.06	105	6.26	< 0.001	** < 0.001**	37
MDS-UPDRS III	−0.29	0.11	105	−2.52	0.013	**0.029**	37

Bold indicates FDR p < .05. FDR: False Discovery Rate; DF: Degrees of freedom; SE: Standard error; OA: Older adults; PD: Parkinson’s disease; ST: Single-task; DT: Dual-task; MDS-UPDRS: Movement Disorders Society-Unified Parkinson’s Disease Rating Scale

### Dual-task costs and prioritization

Secondary analyses revealed relationships between a higher prefrontal activity and higher dual-task cost of walking speed (β = 0.25, T = 2.90, p = 0.02) and Stroop reaction time (β = 0.27, T = 3.10, p = 0.01) in PD, but not in older adults (Table 3). Priority was not significant in either group.

**Table 3:** fNIRS linear model results for aim 3: relating prefrontal cortex activity to dual-task costs and prioritization

Covariate	Beta	SE	DF	T	p	FDR p	N
Group: OA							
DT cost walking speed	−0.16	0.10	127	−1.57	0.120	0.203	44
DT cost Stroop reaction time	−0.24	0.14	124	−1.69	0.094	0.172	43
Priority	0.03	0.11	124	0.29	0.771	0.795	43
Group: PD							
DT cost walking speed	0.25	0.09	102	2.90	0.005	**0.019**	36
DT cost Stroop reaction time	0.27	0.09	105	3.10	0.002	**0.012**	37
Priority	−0.13	0.09	102	−1.55	0.123	0.203	36

Bold indicates FDR p < .05. Only partial results of models for each aim are shown, with full models for each aim located in supplementary Table 2. Models also control for task condition, age and disease severity

FDR: False Discovery Rate; DF: Degrees of freedom; SE: Standard error; OA: Older adults; PD: Parkinson’s disease; DT=Dual-task

### Moderation of executive function

**Fig. 4:**
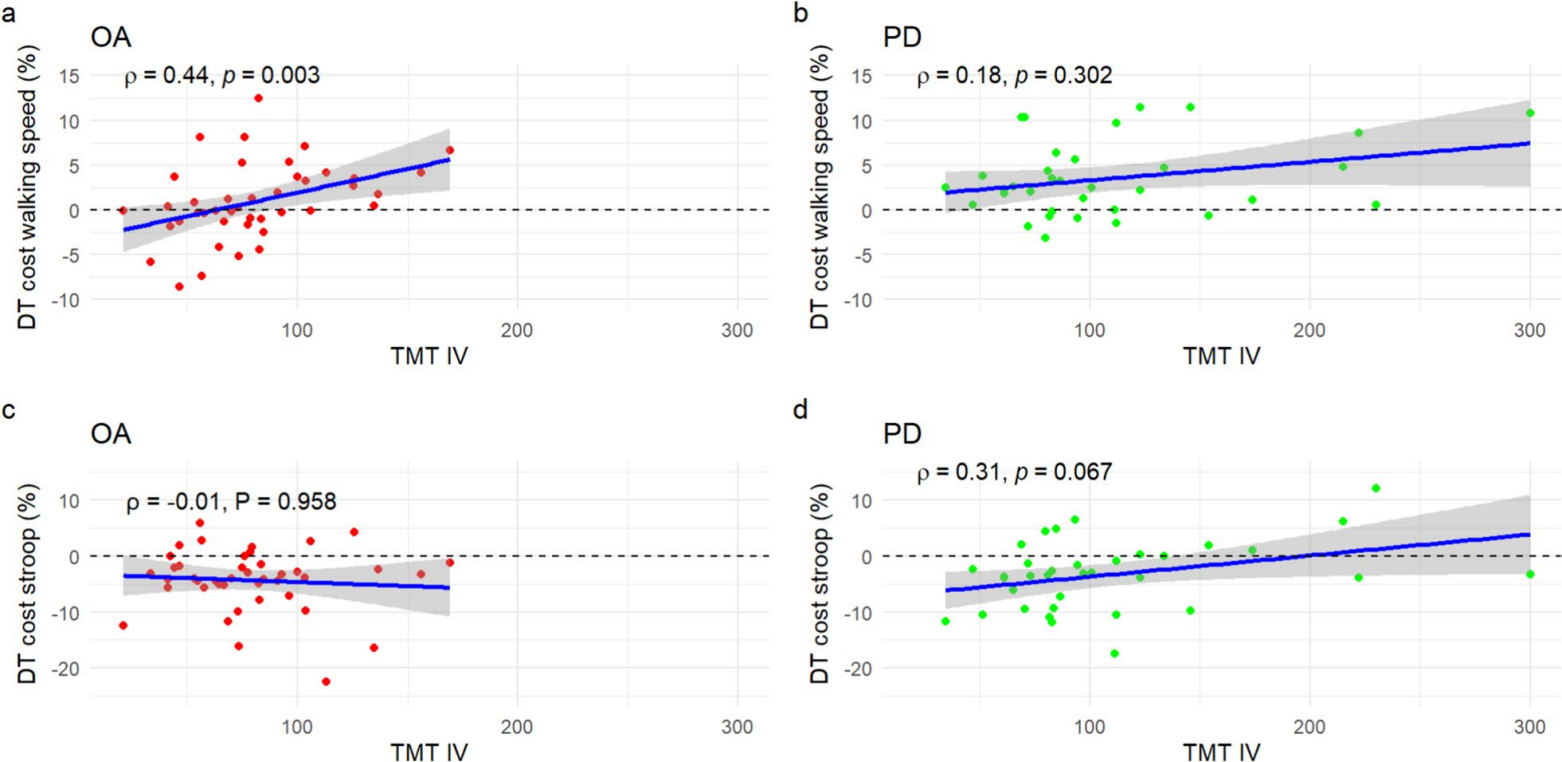
Correlations between trail-making test (TMT) IV and dual-task (DT) costs. **a** TMT IV and walking speed DT cost in OA; **b** TMT IV and walking speed DT cost in PD; **c** TMT IV and Stroop reaction time DT cost in OA; **d** TMT IV and Stroop reaction time DT cost in PD. OA: Older adults; PD: Parkinson’s disease; ST: Single-task; DT: Dual-task

There was a significant positive correlation of moderate strength between executive function (TMT IV completion time) and walking speed dual-task cost in older adults (ρ = 0.44, p < 0.01) (Fig. 4 a).

The moderation analysis however detected no moderation effects (Table 4). In the PD group, both TMT IV completion time (β = −0.42, T = −5.57, p < 0.01) and step time variability (β = 0.38, T = 4.99, p < 0.01) were related to prefrontal cortex increase, but there was no interaction effect between them. As in the first model involving step time variability, a higher step time variability was related to a higher prefrontal activity. A better executive ability (faster TMT IV completion time) was related to a higher prefrontal activity.

**Table 4:** fNIRS linear model results for aim 4: moderation of executive ability in prefrontal cortex activity

Covariate	Beta	SE	DF	T	p	FDR p	N
Group: OA							
Model: R square = 0.162, R square (adjusted) = 0.106, F = 2.896, p < 0.001							
ST_stand condition	−0.28	0.14	121	−1.95	0.053	0.093	43
ST_walk condition	1.06	0.15	121	6.98	< 0.001	** < 0.001**	43
DT_walk condition	1.49	0.17	121	8.94	< 0.001	** < 0.001**	43
Age	0.43	0.33	121	1.31	0.193	0.205	43
Education	0.19	0.10	121	1.93	0.056	0.093	43
Step time variability	−0.26	0.15	121	−1.67	0.097	0.126	43
Step time variability:TMT IV (interaction effect)	0.41	0.27	121	1.52	0.130	0.158	43
TMT IV	0.32	0.18	121	1.74	0.084	0.119	43
Group: PD							
Model: R square 0.198, R square (adjusted) 0.122, F 2.619, p < 0.001							
ST_stand condition	0.29	0.15	96	1.99	0.049	0.093	35
ST_walk condition	0.30	0.16	96	1.90	0.060	0.093	35
DT_walk condition	0.48	0.17	96	2.82	0.006	**0.020**	35
Age	0.43	0.30	96	1.42	0.158	0.179	35
Education	−0.35	0.14	96	−2.49	0.015	**0.041**	35
Step time variability	0.38	0.08	96	4.99	< 0.001	** < 0.001**	35
Step time variability:TMT IV (interaction effect)	0.04	0.06	96	0.60	0.551	0.551	35
TMT IV	−0.42	0.07	96	−5.57	< 0.001	** < 0.001**	35
MDS-UPDRS III	−0.25	0.13	96	−1.97	0.052	0.093	35

Bold indicates FDR p < .05. FDR: False Discovery Rate; DF: Degrees of freedom; SE: Standard error; OA: Older adults; PD: Parkinson s disease; ST: Single-task; DT: Dual-task; TMT: Trail-making test; MDS-UPDRS: Movement Disorders Society-Unified Parkinson’s Disease Rating Scale

Education was also related to prefrontal cortex increase in the PD group (β = −0.35, T = −2.49, p = 0.01), with the coefficient indicating that fewer years of education were related to a higher prefrontal activity.

### Model validation

Linear model residuals had a slightly fat-tailed distribution for the older adult group models, making the results in the older adult group slightly anticonservative. Distributions in the PD group models were more normally distributed.

Two outlier subjects were identified in the older adult group and one in the PD group via the RemoveOutlierSubjects function, corresponding to influential outlier points in the residual-leverage plots. When re-running the analysis without these subjects, all effects of covariates remained robust, with some condition effects (ST_stand for OA in aim 1, DT_walk for PD in aim 2, ST_stand for OA in aim 3) that were around FDR-adjusted p < 0.05 changing significance.

## Discussion

This study captured behavioral (walking and cognition) and prefrontal cortical activation patterns of older adults and people with PD during single-task and dual-task walking conditions. Overall, these results support a link between gait automaticity and prefrontal cortex activity in people with PD. Further, across-group analyses provided modest evidence of over-prioritization of the cognitive task during dual-task walking in people with PD compared to older adults. Interpretation of these and other results are discussed below in turn.

### Task performance and dual-task effects

Similar to earlier observations [34], the PD group had a larger degree of cognitive-motor interference, with a larger dual-task cost on walking speed (Table 1) compared to the older adult group (4.35% vs 0.92%). The PD group also had a higher step time variability during dual-task walking, although the group difference in dual-task cost on step time variability was not significant. Performance of the cognitive task was similar in both groups, both during single and dual-task.

Plotted in quadrants (Fig. 1), the dual-task effects seem to support the posture-second hypothesis [8], where the majority of the PD group end up in the cognitive priority trade-off quadrant (52.8%). While relatively fewer older adults were in this quadrant (41.9%), this may have been because some older adults exhibited too little motor dual-task effects to reach the cognitive priority trade-off quadrant, with several staying around the zero line. As such, for the older adult group it might not be a question of priority during dual-tasking, but instead capacity to maintain motor performance during dual-tasking or even improve in performance. The type of task likely plays a role in these effects. The Stroop task used in this study involves both inhibition of prepotent responses [63] and reaction speed. Interestingly, a study on dual-tasking in older adults showed the least amount of motor interference with a reaction speed task compared to verbal fluency, counting backwards, or list recall tasks [26]. At the same time, a meta-analysis of dual-task studies in PD, although lacking reaction speed tests, has shown significant motor interference regardless of task type [52]. Presumably, reduced automaticity in PD reduces the capacity to maintain performance even with this type of task.

### Comparison between groups

We assessed the effect of PD on the change in prefrontal activity (compared to rest) during standing auditory Stroop, single-task walking, and dual-task walking (Fig. 2). Results indicate that the older adult group had a larger increase in prefrontal activity in the left hemisphere during single and dual-task walking compared to the PD group. Under-recruitment of neural resources in people with PD including frontal regions has also been observed during working memory tasks [19], and prefrontal connectivity in PD has been found to be abnormal during visuospatial working memory tasks [29].

An important ongoing discussion in the literature regarding abnormal activation patterns in aging and neurological disease is whether these abnormal patterns reflect compensation [9, 11, 53] or conversely changes in neural efficiency or dedifferentiation [36]. Some studies find concurrent evidence for both phenomena in different brain regions [18]. Our results, focused on the prefrontal region, are discussed in terms of compensation. However, it should be noted that a more comprehensive view could be achieved through broader coverage of brain regions, connectivity between these, and structural neuroimaging.

Our findings partially support the CRUNCH model and trajectories of neural compensation in healthy and pathological aging [9]. Compensatory neural activity increases with aging and structural degeneration until a “tipping point” in pathological aging, where the same degree of compensatory activity is no longer possible which leads to task performance decrease [9]. It could be possible that the participants in our PD group have passed such a “tipping point”, although a parametric modulation of task demand along with a measure of structural degeneration [9] would be required in order to fully support this idea. Clinically, it might be interesting to determine where such a “tipping point” is in order to target interventions where functional neural compensation can still be used, or find other strategies to deal with task demand (such as cueing in PD). However, this would require a much larger cohort of participants within different age ranges as compared to the current study.

Older adults had more activity compared to the PD group in the left hemisphere of the prefrontal cortex during single and dual-task walking. Studies investigating lateralization of prefrontal cortex activity during single and dual-task walking have been mixed. While one study found that dual-task gait performance correlated with left and middle prefrontal cortex activation in older adults [62], another study found that right dorsolateral prefrontal cortex activity was associated with intact dual-task performance in older adults [31]. The Hemispheric Asymmetry Reduction in Older Adults (HAROLD) model [10], based on the observation that older adults tend to show bilateral activity compared to younger adults, could be relevant to these observations. If the bilateral activity indicates successful compensation before the “tipping point” discussed above, the reduced degree of compensatory activity after the “tipping point” might be localized to one hemisphere. Larger and specifically focused studies would be required in order to properly test such an idea.

### Loss of gait automaticity relates to increased prefrontal cortex activity in PD

To test the assumption that prefrontal cortex activity occurs to compensate for a loss of gait automaticity, we related the participant’s change in prefrontal activity compared to rest, during both single-task and dual-task conditions, to three different quantifications of gait automaticity: step time variability as well as motor and cognitive dual-task costs.

First, we considered an increase in step time variability to reflect a loss of gait automaticity [24]. The linear model results (Table 2) indicate that in people with PD, a loss of gait automaticity relates to an increase in prefrontal activity. This could also be seen in the correlation between prefrontal activity and step time variability (Fig. 3). This was however not the case in the older adult group, where age was a significant covariate.

For the other quantifications of automaticity (Table 3), the same pattern emerges: an increase in dual-task costs, indicative of a loss of gait automaticity, relates to a higher prefrontal activity in the PD group. Again, this was not the case in the older adult group. This was surprising given our assumption that in the CRUNCH model, the compensatory activity occurs to compensate for a loss of gait automaticity in both older adults and PD. An earlier study in older adults found, as in our study, that age was related to prefrontal activity [47], but that study also found a connection to gait variability, as would be expected. In this study, while the older adults seem to have more prefrontal activity during walking, it is related to different factors compared to the PD group. The older adults could also maintain their performance better during dual-tasking. It could be possible that the automaticity loss in our older adult sample was relatively small, and that the measured prefrontal activity related to performance in other ways. In fact, other studies have found that reduced gait variability is associated with an increased level of prefrontal activity while maintaining gait performance in older adults [1]. Thus, prefrontal activity in older adults might not just occur to cope with a reduced gait automaticity but signify a more general mechanism to cope with task demand in the face of age-related changes in the brain [21].

One possible mechanism specific to compensating for reduced gait automaticity is mediation via the cholinergic system [40, 50]. A recent electroencephalography (EEG) study found an association between reduced alpha reactivity, an indicator of cholinergic system integrity, and gait variability in PD [40]. Combined EEG-fNIRS studies would have the possibility of testing this mechanism in a single study. Another important piece of evidence specific to compensation for reduced locomotor automaticity could be connectivity between the prefrontal cortex, supplementary motor area (SMA) and the basal ganglia [45]. While deeper regions cannot be studied with fNIRS, connectivity between the prefrontal cortex and the SMA would add valuable information on the balance between goal-directed and automatic movement. Especially since recent meta-analyses show that dual-task ability can be improved in people with PD with certain types of training [32], these mechanisms might be interesting to study in interventions.

Dopaminergic therapy in PD influences connectivity in cortical sensorimotor networks and motor networks, reducing abnormal increases in connectivity to several regions of the brain including frontal structures [4]. Thus, it is possible that the levodopa equivalent daily dose (LEDD) would influence the results in the compensatory relationship. While we had some participants missing LEDD data, the model in aim 2 was re-run controlling for LEDD, and we found that the same relationship between prefrontal activity and step time variability remained (supplementary Table 3).

### Executive function did not moderate the automaticity-prefrontal activity relationship

We explored the connection to executive ability in the last aim of the study, investigating whether executive ability (in terms of TMT IV) might act as a moderator in the relationship between a loss of automaticity and prefrontal activity. However, the moderation analysis (Table 4) showed no such moderation effects. Although given the large number of samples required for robust moderation analyses, this study might simply be underpowered to detect such effects. It should also be noted that the groups might have performed similarly in terms of executive function: while TMT II and TMT IV were slower for the PD group compared to the older adults, the contrast between them (TMT IV – TMT II) was similar, indicating that the seemingly worse result in TMT IV might have been due to motor slowness in the PD group.

However, while there was no interaction effect between TMT IV and step time variability in their relationship to prefrontal activity, there were fixed effects of both covariates on their own (Table 4), at least in the PD group. For PD, a better executive ability (shorter TMT IV time) was related to increased prefrontal activity (β = −0.42, T = −5.57, p < 0.01). This might indicate that executive ability does play some role, something that is also supported by earlier studies finding association between executive function and activation in Brodmann area 10 [42]. Another study also found a positive association between cognitive task performance and functional connectivity to the dorsolateral prefrontal cortex from several other regions [7].

The direction of the association was different between the groups in this study: while the effect was not significant for older adults (β = 0.32, T = 1.74, p = 0.119), the effect was in the opposite direction. An earlier study in older adults also found that executive ability had a negative correlation to prefrontal activity [47]. However, further and larger studies are required to determine precisely what role cognitive ability has in the relationship to prefrontal activity during walking and dual-task walking, whether it is moderation or mediation or some other role.

## Limitations

Although cap size was chosen for participants, anatomical registration with a 3D digitizer or photogrammetry methods [20] was not performed, so cap positioning could have deviated somewhat between participants. Additionally, while short channels were used to filter out superficial signal, supplementary physiology like respiration [27] was not captured which could have allowed for a better understanding of the effects of breathing during the auditory Stroop, for example.

The montage used was only 8 × 8. To fully understand patterns of compensation in terms of upregulation, selection and reorganization, studies using whole-brain and denser fNIRS would be useful. Additionally, to better situate the analysis in the CRUNCH model, parametric modulation of task demand and measures of structural degeneration [9] would be good to have in the future.

Finally, the studied PD sample had an overall mild disease severity, with the majority being classified as Hoehn & Yahr stage 2, with a balance score indicating good balance performance. This study might therefore have missed effects present in more severe disease. For example, there was little presence of freezing noted during the walking experiment, which influences prefrontal cortex activity [14].

## Conclusion

Loss of gait automaticity in PD is linked to increased prefrontal activity, possibly reflecting compensatory mechanisms. In older adults, prefrontal activity during walking and dual-task walking is mainly influenced by age.

## Supplementary Information

Supplementary Material 1.

Supplementary Material 2.

Supplementary Material 3.

Supplementary Material 4.

Supplementary Material 5.

Supplementary Material 6.

## Data Availability

All code is available via https:/github.com/alkvi/fnirs_stroop_study and the open lab notebook at https:/alkvi.github.io/fnirs_stroop_study. The original data are not publicly available due to Swedish/EU law, but is located with restricted access in a central repository (10.48723/vscr-eq07), where sharing will be regulated via a data transfer and user agreement upon a reasonable request.
