# Supplementary material for: Exploring gait automaticity and prefrontal brain activity during single and dual-task walking in aging and Parkinson’s disease
Source: J Neuroeng Rehabil. 2026 Jan 5;23:41. doi: 10.1186/s12984-025-01864-w (PMC12849653; doi:10.1186/s12984-025-01864-w)
Supplement: Supplementary file 1 — Supplementary Material 1. [file 12984_2025_1864_MOESM1_ESM.docx]

**Missing data and outlier handling**

49 OA subjects and 42 PD subjects were collected.

In the OA group:

- 1 OA subject was excluded after re-evaluating exclusion criteria
- 2 OA subjects were missing gait data due to data loss during protocol
- 2 OA subjects were identified as outliers in gait data due to unexpected behavior during the protocol (e.g., sensor falling off participant)
- 1 OA subjects were identified as outliers in auditory Stroop data (not understanding the Stroop task)

In the PD group:

- 1 PD subject was missing due to data loss
- 1 PD subject was missing due to not completing protocol
- 1 PD subject was excluded for not following protocol
- 2 PD subjects were missing MDS-UPDRS data

Only remaining subjects with full gait data were used.

For models involving only gait data, 10.2% of OA and 11.9% of PD were not included.
