# Supplementary figures and images for: Exploring gait automaticity and prefrontal brain activity during single and dual-task walking in aging and Parkinson’s disease
Source: J Neuroeng Rehabil. 2026 Jan 5;23:41. doi: 10.1186/s12984-025-01864-w (PMC12849653; doi:10.1186/s12984-025-01864-w)

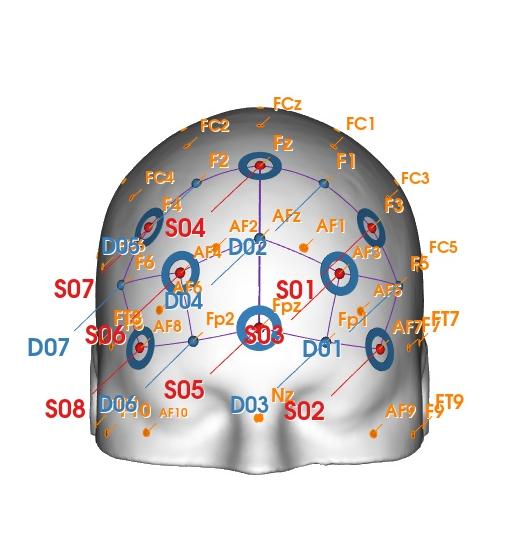

Supplement: Supplementary file 2 — Supplementary Material 2. [file 12984_2025_1864_MOESM2_ESM.jpeg]

ST\_stand

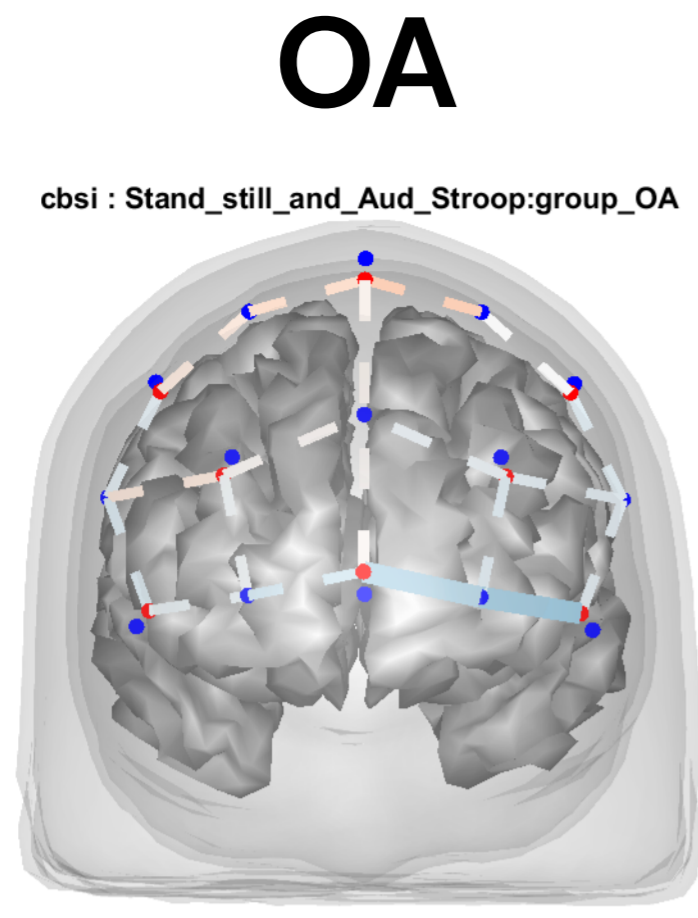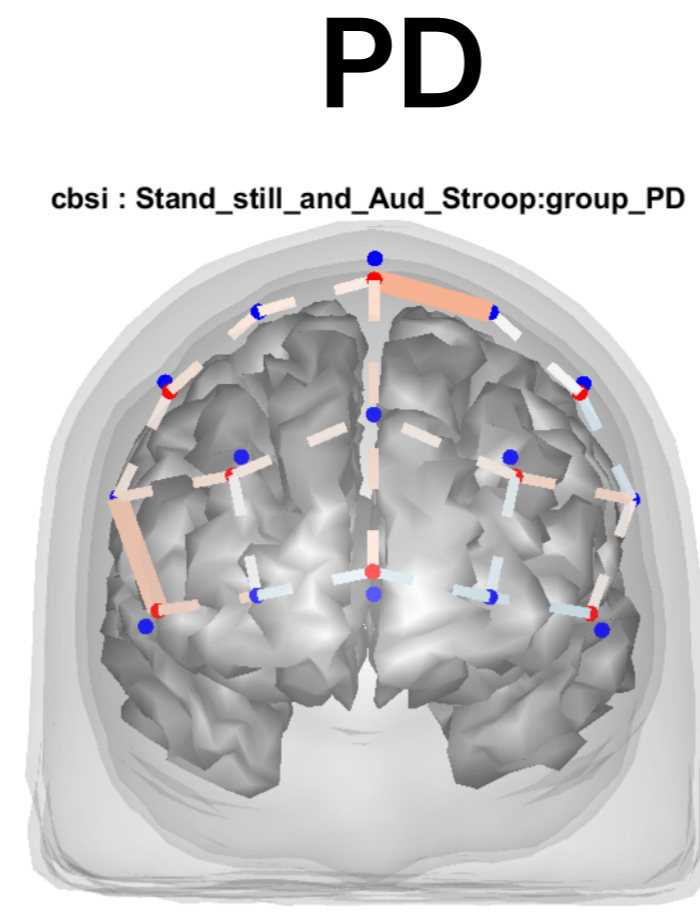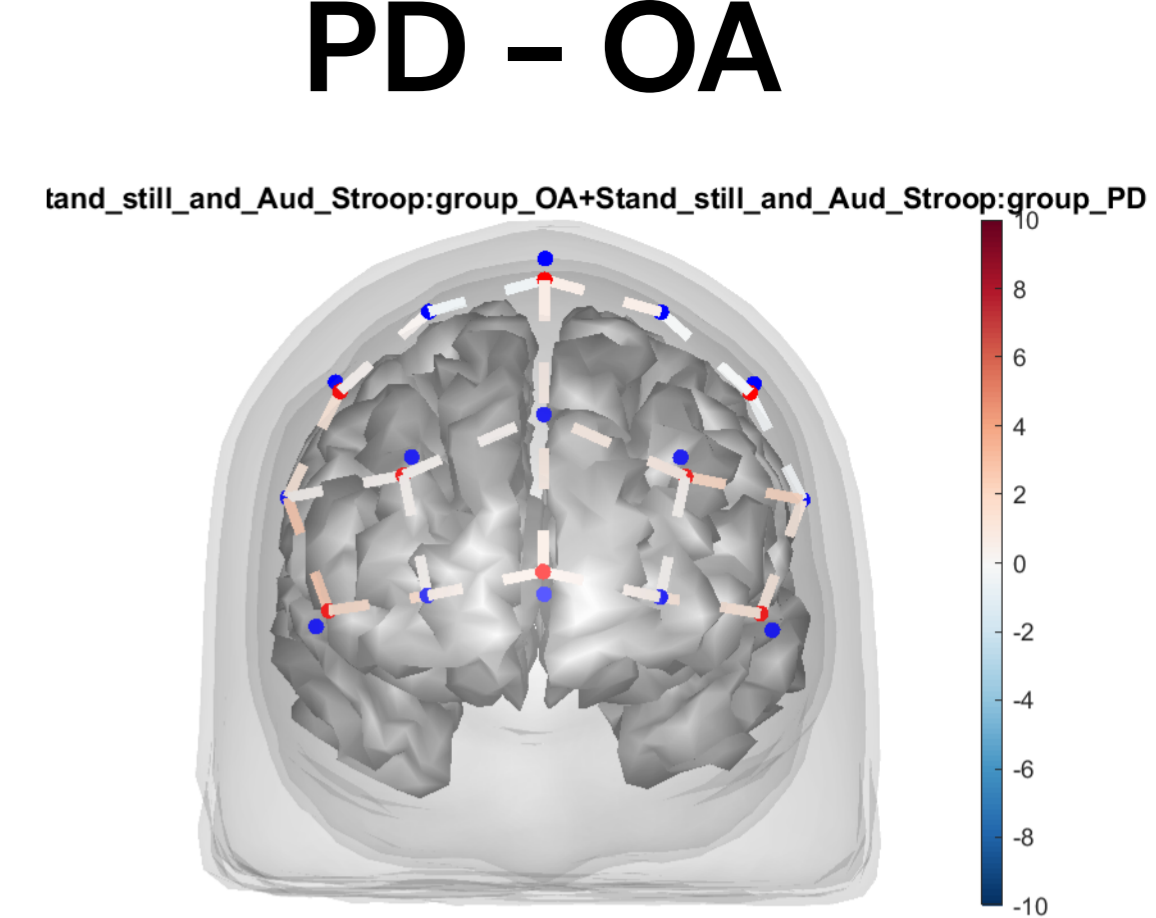

ST\_walk

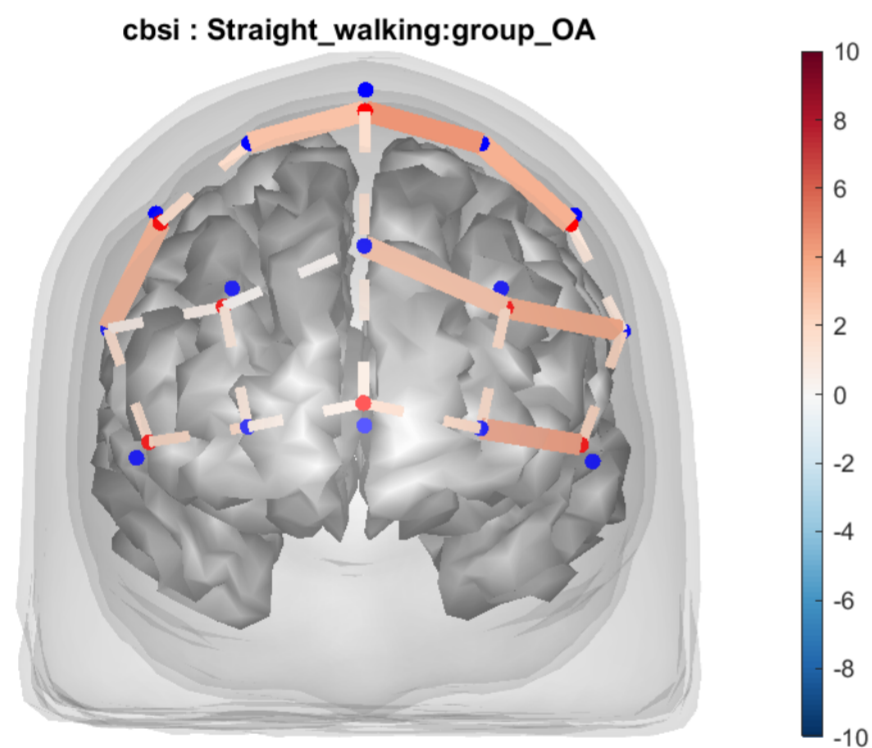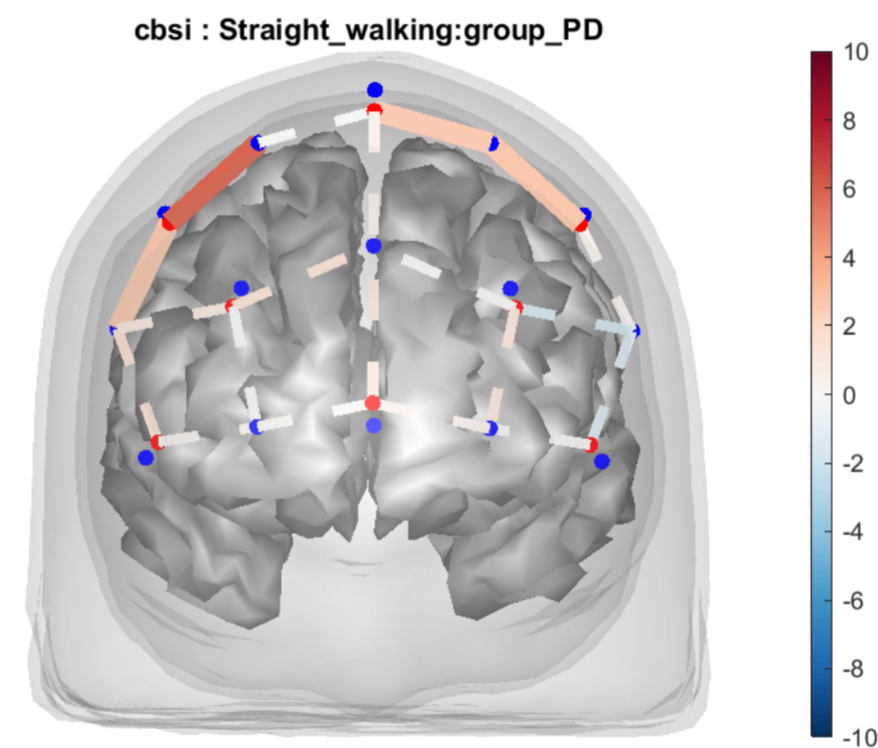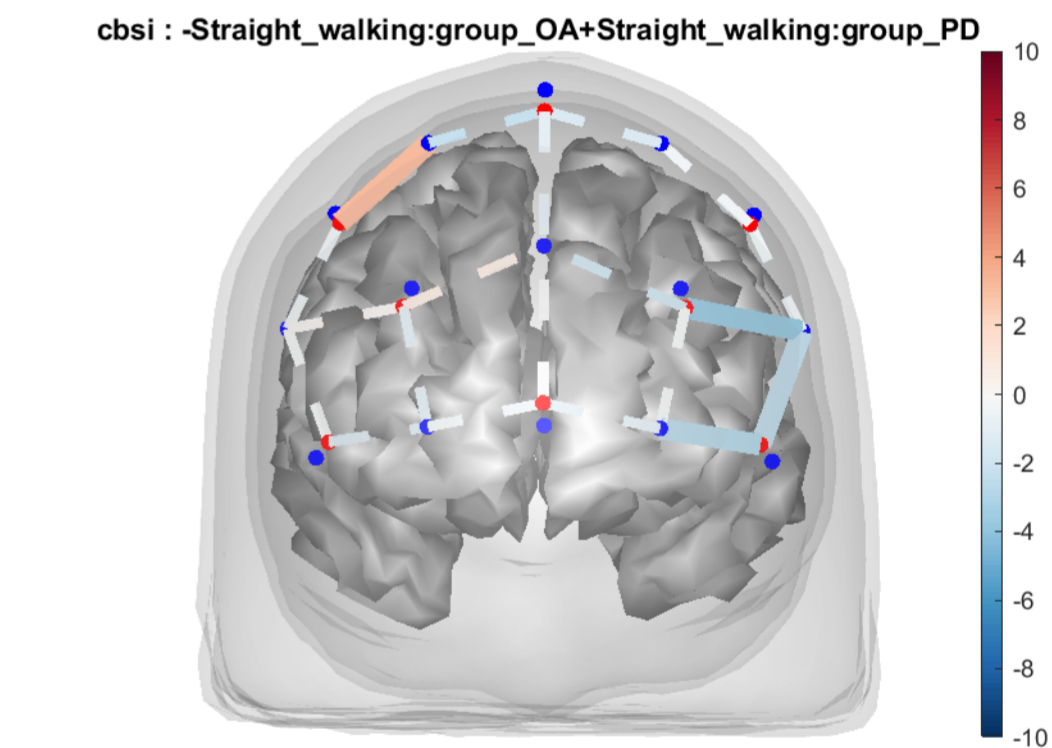

DT\_walk

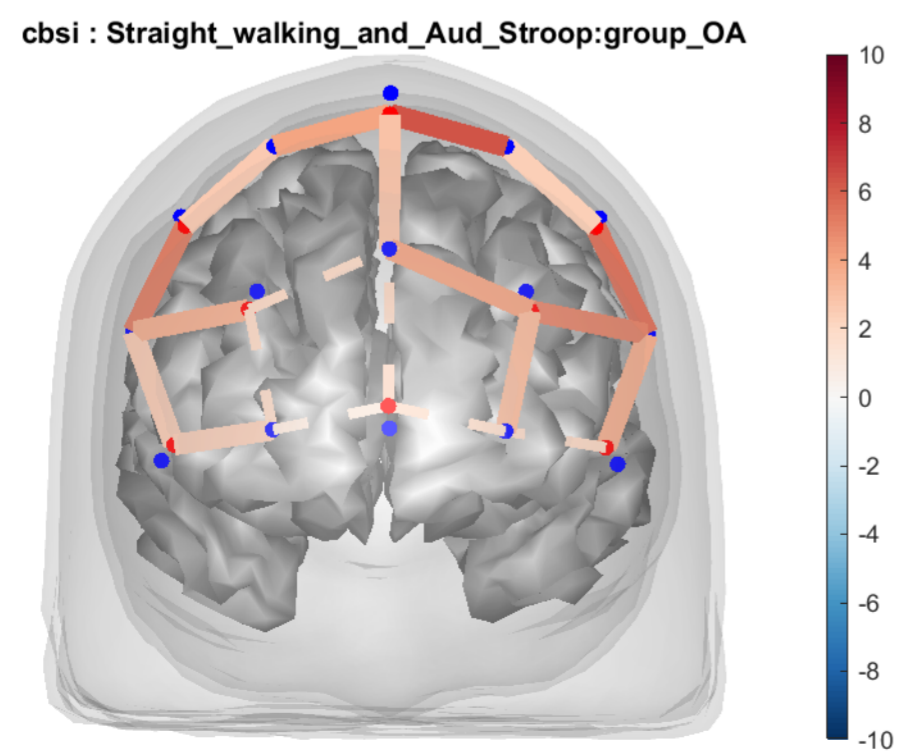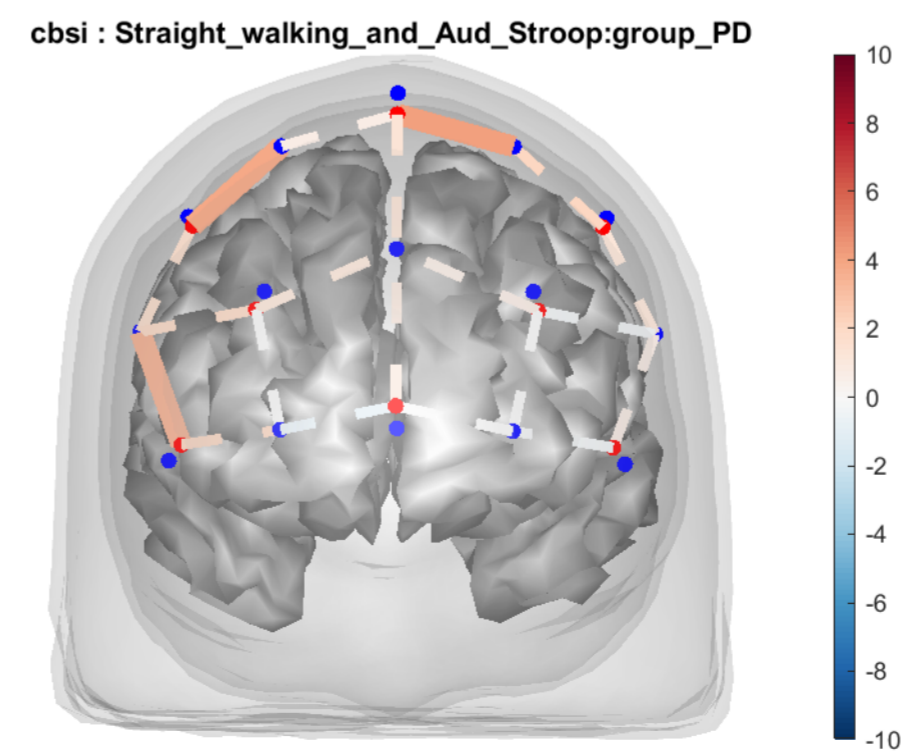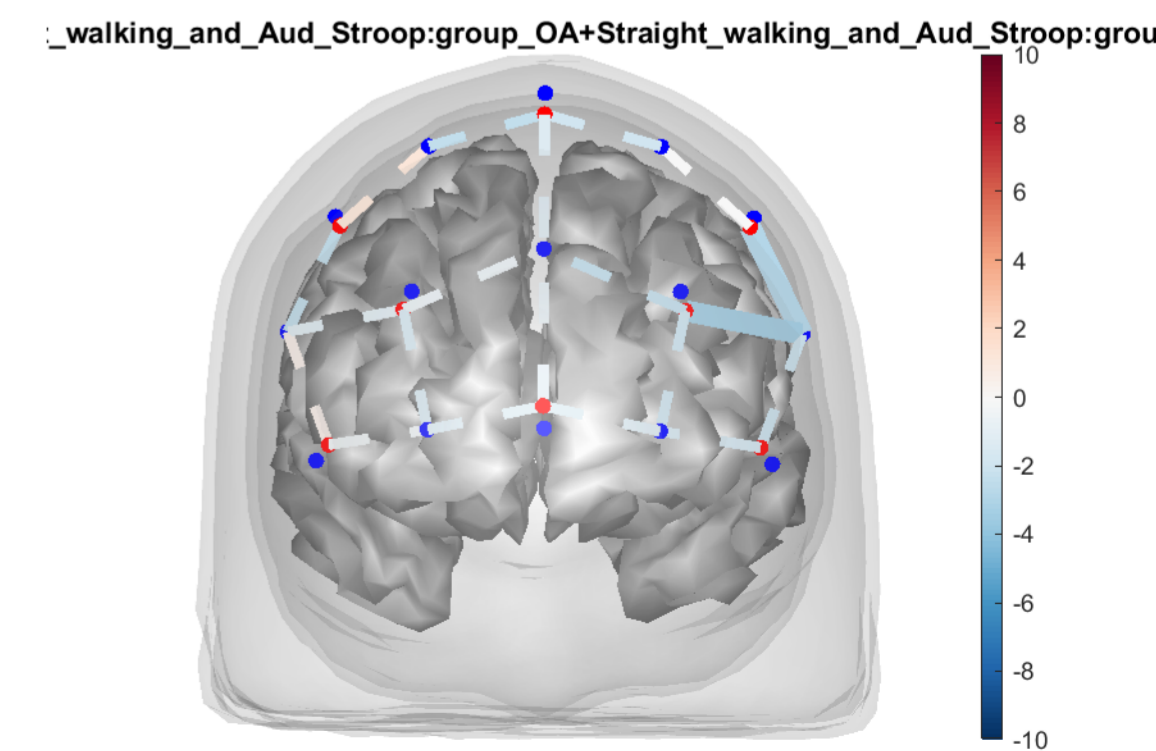

Supplement: Supplementary file 3 — Supplementary Material 3. [file 12984_2025_1864_MOESM3_ESM.pdf]
