# Supplementary material for: Exploring gait automaticity and prefrontal brain activity during single and dual-task walking in aging and Parkinson’s disease
Source: J Neuroeng Rehabil. 2026 Jan 5;23:41. doi: 10.1186/s12984-025-01864-w (PMC12849653; doi:10.1186/s12984-025-01864-w)
Supplement: Supplementary file 5 — Supplementary Material 5. [file 12984_2025_1864_MOESM5_ESM.docx]

| **Condition** | **Beta** | **SE** | **DF** | **T** | **p** | **p_adjusted** | **group** | **Aim** | **N** |
| --- | --- | --- | --- | --- | --- | --- | --- | --- | --- |
| **Group: PD** Model: R square 0.135, R square (adjusted) 0.0841, F 2.639, p <.001 | | | | | | | | | |
| UPDRS 3 Motor | −0.22 | 0.11 | 102 | −2.00 | 0.048 | 0.103 | PD | Aim 2_1 | 36 |
| ST_stand | 0.19 | 0.14 | 102 | 1.34 | 0.184 | 0.233 | PD | Aim 2_1 | 36 |
| ST_walk | 0.17 | 0.15 | 102 | 1.10 | 0.275 | 0.324 | PD | Aim 2_1 | 36 |
| DT_walk | 0.36 | 0.17 | 102 | 2.17 | 0.032 | 0.075 | PD | Aim 2_1 | 36 |
| age | −0.20 | 0.26 | 102 | −0.78 | 0.436 | 0.479 | PD | Aim 2_1 | 36 |
| **DT cost walking speed** | **0.25** | **0.09** | **102** | **2.90** | **0.005** | **0.019** | **PD** | **Aim 2_1** | **36** |
| **Group: OA** Model: R square 0.118, R square (adjusted) 0.0836, F 3.379, p <.001 | | | | | | | | | |
| ST_stand | −0.20 | 0.14 | 127 | −1.45 | 0.151 | 0.207 | OA | Aim 2_1 | 44 |
| **ST_walk** | **1.00** | **0.15** | **127** | **6.70** | **<.001** | **<.001** | **OA** | **Aim 2_1** | **44** |
| **DT_walk** | **1.47** | **0.16** | **127** | **8.91** | **<.001** | **<.001** | **OA** | **Aim 2_1** | **44** |
| **age** | **0.62** | **0.24** | **127** | **2.61** | **0.010** | **0.033** | **OA** | **Aim 2_1** | **44** |
| DT cost walking speed | −0.16 | 0.10 | 127 | −1.57 | 0.120 | 0.203 | OA | Aim 2_1 | 44 |

| **Condition** | **Beta** | **SE** | **DF** | **T** | **p** | **p_adjusted** | **group** | **Aim** | **N** |
| --- | --- | --- | --- | --- | --- | --- | --- | --- | --- |
| **Group: PD** Model: R square 0.132, R square (adjusted) 0.0823, F 2.656, p <.001 | | | | | | | | | |
| UPDRS 3 Motor | −0.12 | 0.12 | 105 | −1.03 | 0.304 | 0.346 | PD | Aim 2_2 | 37 |
| ST_stand | 0.21 | 0.14 | 105 | 1.47 | 0.145 | 0.207 | PD | Aim 2_2 | 37 |
| ST_walk | 0.27 | 0.15 | 105 | 1.78 | 0.078 | 0.151 | PD | Aim 2_2 | 37 |
| **DT_walk** | **0.44** | **0.17** | **105** | **2.63** | **0.010** | **0.033** | **PD** | **Aim 2_2** | **37** |
| age | 0.09 | 0.25 | 105 | 0.35 | 0.731 | 0.778 | PD | Aim 2_2 | 37 |
| **DT cost Stroop reaction time** | **0.27** | **0.09** | **105** | **3.10** | **0.002** | **0.012** | **PD** | **Aim 2_2** | **37** |
| **Group: OA** Model: R square 0.115, R square (adjusted) 0.0799, F 3.211, p <.001 | | | | | | | | | |
| **ST_stand** | **−0.36** | **0.14** | **124** | **−2.58** | **0.011** | **0.033** | **OA** | **Aim 2_2** | **43** |
| **ST_walk** | **0.93** | **0.15** | **124** | **6.21** | **<.001** | **<.001** | **OA** | **Aim 2_2** | **43** |
| **DT_walk** | **1.31** | **0.17** | **124** | **7.95** | **<.001** | **<.001** | **OA** | **Aim 2_2** | **43** |
| age | 0.47 | 0.24 | 124 | 1.98 | 0.050 | 0.103 | OA | Aim 2_2 | 43 |
| DT cost Stroop reaction time | −0.24 | 0.14 | 124 | −1.69 | 0.094 | 0.172 | OA | Aim 2_2 | 43 |

| **Condition** | **Beta** | **SE** | **DF** | **T** | **p** | **p_adjusted** | **group** | **Aim** | **N** |
| --- | --- | --- | --- | --- | --- | --- | --- | --- | --- |
| **Group: PD** Model: R square 0.138, R square (adjusted) 0.0871, F 2.708, p <.001 | | | | | | | | | |
| UPDRS 3 Motor | −0.17 | 0.11 | 102 | −1.51 | 0.135 | 0.205 | PD | Aim 2_3 | 36 |
| ST_stand | 0.21 | 0.14 | 102 | 1.50 | 0.137 | 0.205 | PD | Aim 2_3 | 36 |
| ST_walk | 0.20 | 0.15 | 102 | 1.30 | 0.198 | 0.242 | PD | Aim 2_3 | 36 |
| DT_walk | 0.39 | 0.17 | 102 | 2.35 | 0.021 | 0.053 | PD | Aim 2_3 | 36 |
| age | −0.03 | 0.26 | 102 | −0.13 | 0.895 | 0.895 | PD | Aim 2_3 | 36 |
| Priority | −0.13 | 0.09 | 102 | −1.55 | 0.123 | 0.203 | PD | Aim 2_3 | 36 |
| **Group: OA** Model: R square 0.115, R square (adjusted) 0.0797, F 3.206, p <.001 | | | | | | | | | |
| **ST_stand** | **−0.34** | **0.14** | **124** | **−2.42** | **0.017** | **0.046** | **OA** | **Aim 2_3** | **43** |
| **ST_walk** | **0.95** | **0.15** | **124** | **6.34** | **<.001** | **<.001** | **OA** | **Aim 2_3** | **43** |
| **DT_walk** | **1.34** | **0.17** | **124** | **8.06** | **<.001** | **<.001** | **OA** | **Aim 2_3** | **43** |
| age | 0.31 | 0.22 | 124 | 1.40 | 0.163 | 0.215 | OA | Aim 2_3 | 43 |
| Priority | 0.03 | 0.11 | 124 | 0.29 | 0.771 | 0.795 | OA | Aim 2_3 | 43 |
