# Supplementary material for: Exploring gait automaticity and prefrontal brain activity during single and dual-task walking in aging and Parkinson’s disease
Source: J Neuroeng Rehabil. 2026 Jan 5;23:41. doi: 10.1186/s12984-025-01864-w (PMC12849653; doi:10.1186/s12984-025-01864-w)
Supplement: Supplementary file 6 — Supplementary Material 6. [file 12984_2025_1864_MOESM6_ESM.docx]

| **Condition** | **Beta** | **SE** | **DF** | **T** | **p** | **q** | **group** | **Aim** | **included_n** |
| --- | --- | --- | --- | --- | --- | --- | --- | --- | --- |
| **UPDRS 3 Motor** | **−0.338** | **0.128** | **86** | **−2.636** | **0.010** | **0.035** | **PD** | **LEDD** | **31** |
| ST_stand | 0.222 | 0.153 | 86 | 1.448 | 0.151 | 0.212 | PD | LEDD | 31 |
| ST_walk | 0.145 | 0.164 | 86 | 0.884 | 0.379 | 0.442 | PD | LEDD | 31 |
| DT_walk | 0.130 | 0.179 | 86 | 0.730 | 0.468 | 0.468 | PD | LEDD | 31 |
| age | 0.591 | 0.289 | 86 | 2.043 | 0.044 | 0.077 | PD | LEDD | 31 |
| **LEDD** | **0.264** | **0.112** | **86** | **2.355** | **0.021** | **0.049** | **PD** | **LEDD** | **31** |
| **Step time variability** | **0.198** | **0.075** | **86** | **2.648** | **0.010** | **0.035** | **PD** | **LEDD** | **31** |
